# Supplementary material for: Directed self-assembly of a xenogeneic vascularized endocrine pancreas for type 1 diabetes
Source: Nat Commun. 2023 Feb 16;14:878. doi: 10.1038/s41467-023-36582-1 (PMC9935529; doi:10.1038/s41467-023-36582-1)
Supplement: Supplementary file 1 — Supplementary Information [file 41467_2023_36582_MOESM1_ESM.pdf]

Supplementary Information

for:

Directed Self-Assembly of a Xenogeneic Vascularized Endocrine Pancreas for Type 1 Diabetes

Antonio Citro *[1], Alessia Neroni [1,2], Cataldo Pignatelli [1], Francesco Campo [1,2], Martina Policardi [1], Matteo Monieri [1], Silvia Pellegrini [1], Erica Dugnani [1], Fabio Manenti [1], Maria Chiara Maffia [1], Libera Valla [1,2,3], Elisabeth Kemter [2,3,4], Ilaria Marzinotto [1], Cristina Olgasi [6], Alessia Cucci [6], Antonia Follenzi [6], Vito Lampasona [1], Eckhard Wolf [2,3,4] and Lorenzo Piemonti [1,2]

Affiliations:

[1] San Raffaele Diabetes Research Institute, IRCCS San Raffaele Scientific Institute, 20132 Milan, Italy.

[2] Università Vita-Salute San Raffaele, Milan, Italy.

[3]Chair for Molecular Animal Breeding and Biotechnology, Gene Center and Department of Veterinary Sciences, LMU Munich, 81377 Munich, Germany.

[4] Center for Innovative Medical Models (CiMM), Department of Veterinary Sciences, LMU Munich, 85764 Oberschleißheim, Germany.

[5] German Center for Diabetes Research (DZD), 85764 Neuherberg, Germany.

[6] Department of Health Sciences, School of Medicine, University of Piemonte Orientale, 28100 Novara, Italy.

The file includes:

Supplementary Figure 1: BOEC gating strategy

Supplementary Figure 2: assessment of VEP ex vivo gene expression

Supplementary Figure 3: assessment of BOEC role in VEP endocrine performance ex vivo

Supplementary Figure 4: DL-NPIs iRFP detection 1 and 9 weeks after implantation

Supplementary Figure 5: assessment of BOEC role in VEP endocrine performance in vivo

Supplementary Figure 6: assessment VEP vascular composition 9 weeks after implantation

Supplementary Figure 7: assessment of VEP vascular and nerve structure 14 weeks after implantation

Supplementary Figure 8: evaluation of NSG residual neutrophils and mono/macrophages VEP infiltration

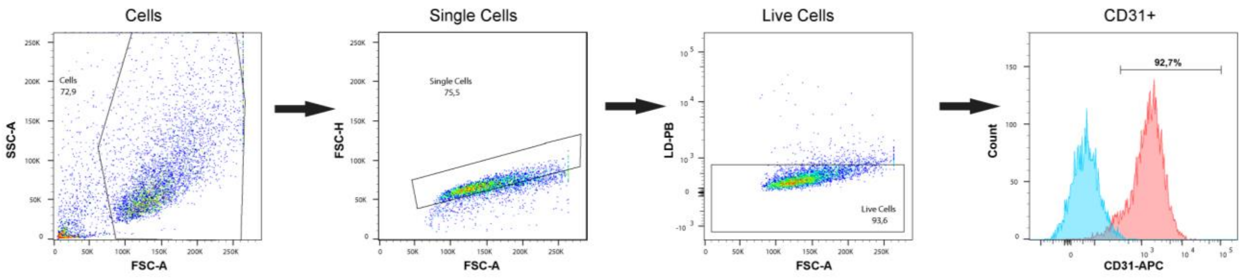

Supplementary Figure 1 – Gating strategy used for flow cytometric analysis of BOEC.

We selected cells by dimension (FSC-A vs SSC-A), then the single cells (FSC-A vs FSC-H) and live cells, stained with a live/dead marker. Finally live cells were evaluated for specific endothelial markers (i.e., CD31- APC)

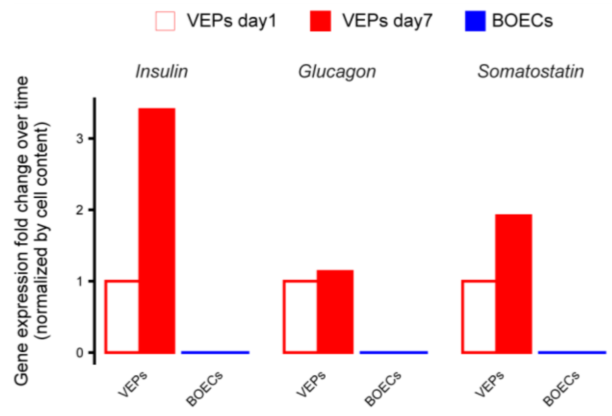

Supplementary Figure 2 – assessment of VEP ex vivo gene expression

Pig insulin, glucagon and somatostatin mRNA levels, expressed as fold change over time of VEP day 1 (white) and day 7 (red). BOEC (blue) were used as internal control. Data were normalized by cell content. Source data are provided as a Source Data file

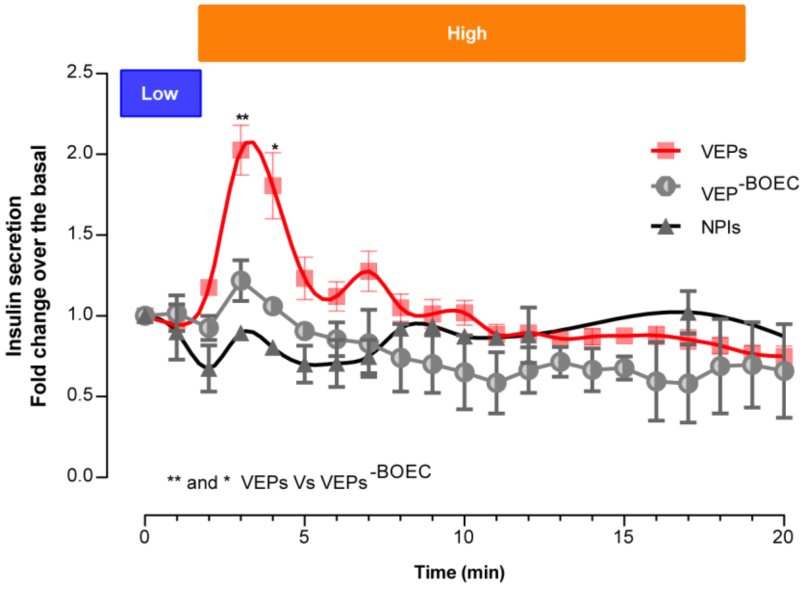

Supplementary Figure 3 – Assessment of BOEC role in VEP endocrine performance

Insulin secretion test of VEPs (red line, n=8 scaffolds) vs. VEPs^{-BOEC} (grey line, n=3 scaffold) and NPIs (black line, n=10 preparations) after 7 days of culture in the presence of low (2mM) and high (20mM) glucose. Values are expressed as fold change over the basal and presented as Mean ± SEM. (VEPs vs VEPs^{-BOEC} ** min 3 p=0.009 and * min 4 p=0.0157, one way ANOVA for multiple comparisons). Source data are provided as a Source Data file

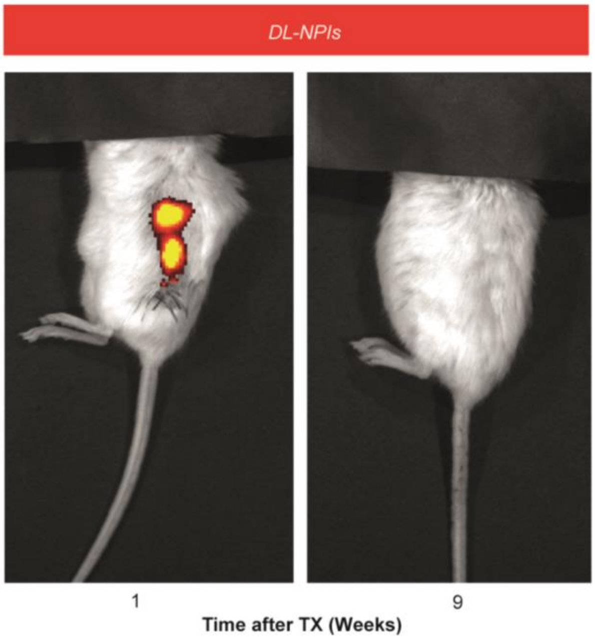

Supplementary Figure 4 - DL-NPIs iRFP detection 1 and 9 weeks after implantation

IVIS detection of the iRFP NPIs signal (detected graft- yellow/orange) in DL-NPIs transplanted at 1 (left panel) and 9 weeks (right panel – no signal) after transplantation.

A

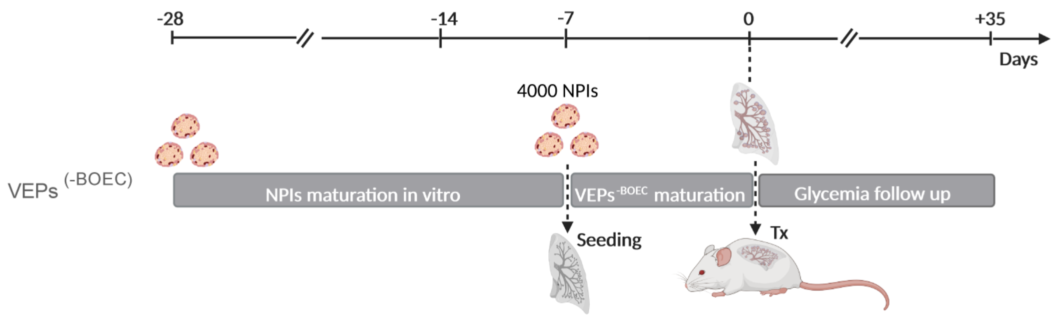

B

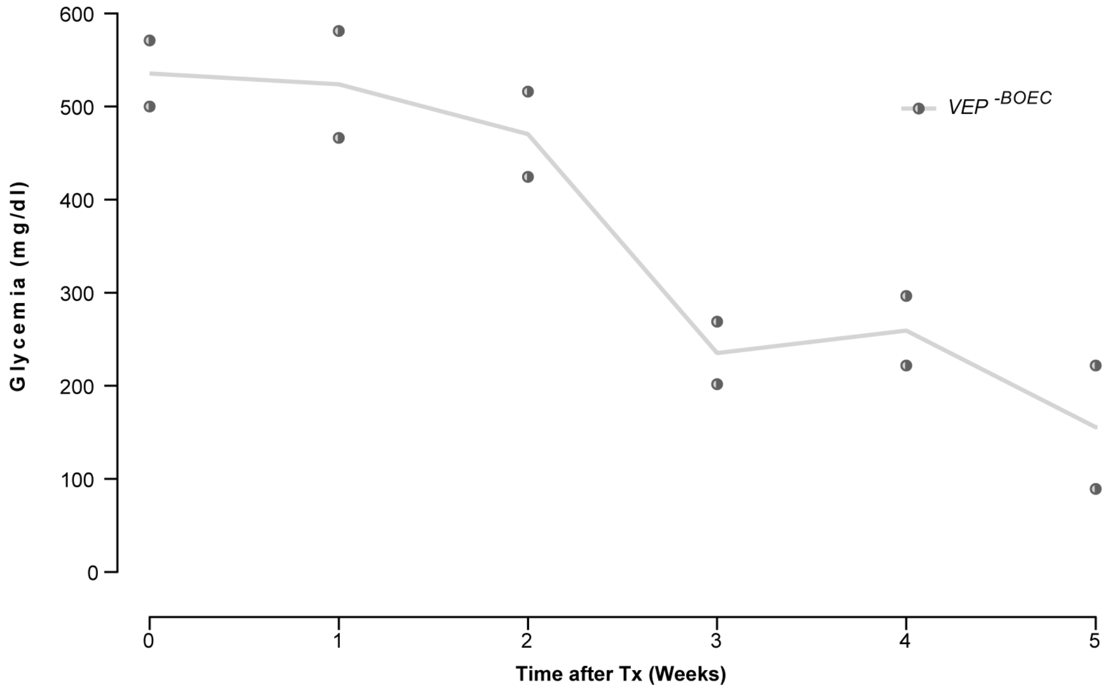

Supplementary Figure 5: Assessment of BOEC role in VEP endocrine performance in vivo

(A) Schematic representation of in vivo VEP^{-BOEC} experimental protocol. Created with Biorender **(B)** Weekly not fasting glycemia profile of VEP^{-BOEC} transplanted mice (grey, n=2) for 5 weeks follow up. Values presented as individual replicates with means connected. Source data are provided as a Source Data file

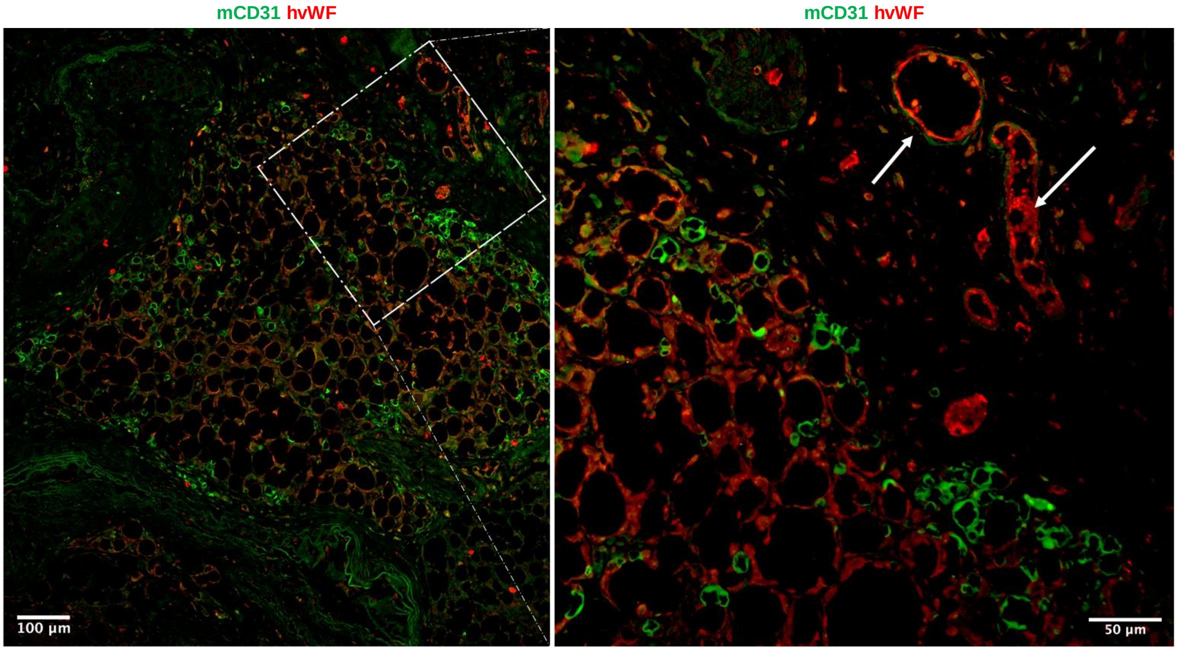

Supplementary Figure 6 - Assessment VEP vascular composition 9 weeks after implantation

Left panel: VEP Immunofluorescence 9 weeks after implantation. Vascular structure was stained for human (von Willebrand factor – hvWF red) and murine (mCD31- green) endothelial cells. **Right panel:** immunofluorescence zoom in from the dashed square from left panel. Image shows vascular transition between VEP human endothelial vascular bed (red) with red blood cells in the vessel lumen (white arrows) and murine endothelial cells (green). Scale bar in μm. Two independent experiments; the representative images were shown.

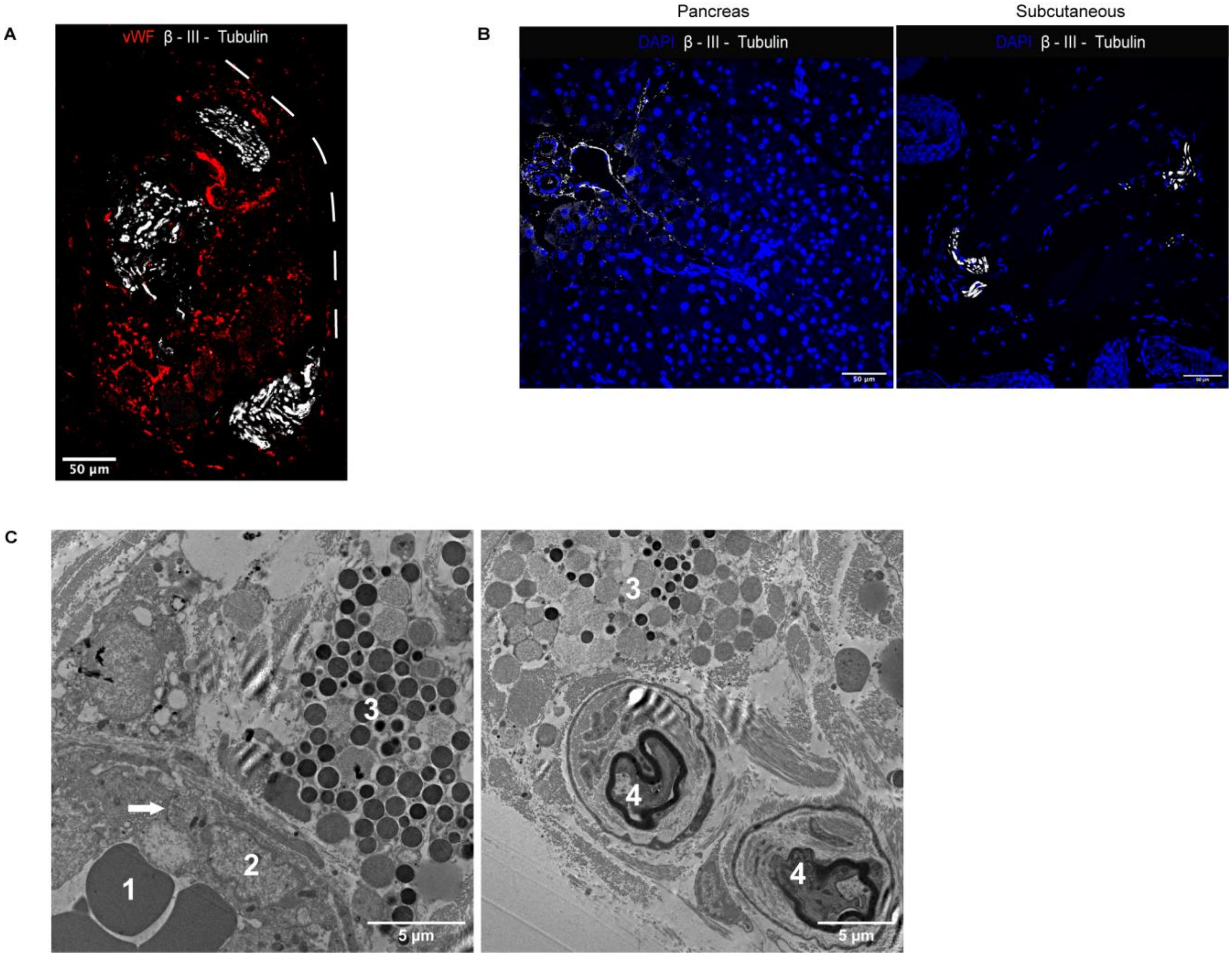

Supplementary Figure 7: assessment of VEP vascular and nerve structure 14 weeks after implantation

(A) VEP Immunofluorescence 14 weeks after implantation. Vascularization and innervation were evaluated with human von Willebrand factor (vWF, red) and murine β III Tubulin (β III Tubulin - white) respectively. White dashed line represents VEP margins. Two independent experiments; the representative images were shown. **(B)** Murine β III Tubulin (β III Tubulin – white, DAPI -blue) immunofluorescence of control pancreas and subcutaneous space from not manipulated NSG tissues. Two independent experiments; the representative images were shown. **(C)** VEP TEM 14 weeks after transplantation showing (1) red blood cells, (2) endothelial cells, (3) endocrine granular cells, (4) myelinated somatic peripheral nerves surrounded by perineurium and tight junction (white arrow). Scale bar in μm. Three independent experiments; the representative images were shown.

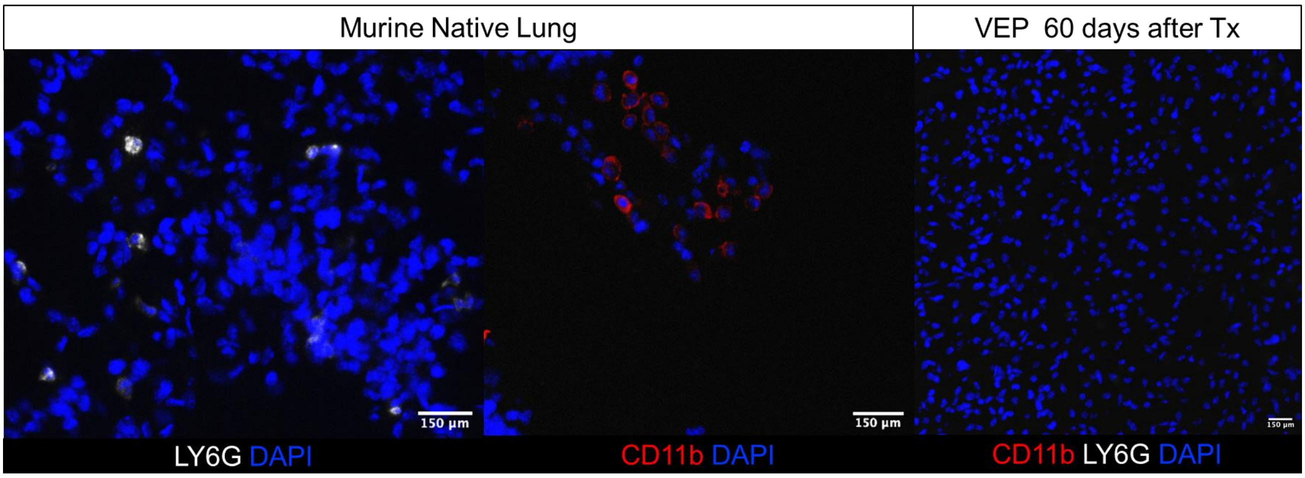

Supplementary Figure 8 –Evaluation of NSG residual neutrophils and mono/macrophages VEP infiltration

Left panel: murine native lung stained, as control, for neutrophils (murine LY6G, white) and macrophage (murine CD11b, red). **Right panel:** 60 days harvested VEP stained for murine LY6G (white) and murine CD11b (red) for murine neutrophil and macrophage. No CD11b nor LY6G positive cells were detected within the analyzed samples. DAPI in blue. Scale bar in µm. Two independent experiments; the representative images were shown.
