# Supplementary material for: Directed self-assembly of a xenogeneic vascularized endocrine pancreas for type 1 diabetes
Source: Nat Commun. 2023 Feb 16;14:878. doi: 10.1038/s41467-023-36582-1 (PMC9935529; doi:10.1038/s41467-023-36582-1)
Supplement: Supplementary file 3 — Reporting Summary [file 41467_2023_36582_MOESM3_ESM.pdf]

nature portfolio

Corresponding author(s): Dr. Antonio Citro
Last updated by author(s): Jan 13, 2023

Reporting Summary

Nature Portfolio wishes to improve the reproducibility of the work that we publish. This form provides structure for consistency and transparency in reporting. For further information on Nature Portfolio policies, see our Editorial Policies and the Editorial Policy Checklist.

Statistics

For all statistical analyses, confirm that the following items are present in the figure legend, table legend, main text, or Methods section.

n/a	Confirmed	
☐	☒	The exact sample size (*n*) for each experimental group/condition, given as a discrete number and unit of measurement
☐	☒	A statement on whether measurements were taken from distinct samples or whether the same sample was measured repeatedly
☐	☒	The statistical test(s) used AND whether they are one- or two-sided<br>*Only common tests should be described solely by name; describe more complex techniques in the Methods section.*
☒	☐	A description of all covariates tested
☐	☒	A description of any assumptions or corrections, such as tests of normality and adjustment for multiple comparisons
☐	☒	A full description of the statistical parameters including central tendency (e.g. means) or other basic estimates (e.g. regression coefficient) AND variation (e.g. standard deviation) or associated estimates of uncertainty (e.g. confidence intervals)
☐	☒	For null hypothesis testing, the test statistic (e.g. *F*, *t*, *r*) with confidence intervals, effect sizes, degrees of freedom and *P* value noted<br>*Give P values as exact values whenever suitable.*
☒	☐	For Bayesian analysis, information on the choice of priors and Markov chain Monte Carlo settings
☒	☐	For hierarchical and complex designs, identification of the appropriate level for tests and full reporting of outcomes
☒	☐	Estimates of effect sizes (e.g. Cohen's *d*, Pearson's *r*), indicating how they were calculated

Our web collection on statistics for biologists contains articles on many of the points above.

Software and code

Policy information about availability of computer code

Data collection	Microsoft Excel 2013 version 15.0.5501.1000<br>EVOS™ XL core AMEX 1000 Imaging System was used to collected optical, phase-contrast images.<br>Confocal Images were capture by Olympus Fluoview 3000rs<br>For in vivo vascular analysis collected images with Leica s9i<br>For TEM we used ThermoFisher Talos L120C electron microscope.<br>For IVIS detection we used Living Image 4.5 (Perkin Elmer).
Data analysis	- Cells were analyzed by flow citometry using FACSCantoII instrument (BD Biosciences) and FlowJo 10.6.2 as software.<br>- Fluorescent analysis were performed using Fiji imageJ software version: 2.1.0/1.53C.<br>- For Statistical analysis we used Graphpad Prism v9 version 9.4.1 (GraphPad Software, http://www.graphpad.com) and R version 4.2.2 (R Core Team (2020) R : A Language and Environment for Statistical Computing. R Foundation for Statistical Computing, Vienna, Austria; rstatix and survminer packages)<br>For RNA and DNA: samples were quantified by the Quantus™ Fluorometer (Promega), using the QuantiFluor® RNA System or the QuantiFluor® ONE dsDNA System, respectively.<br>- For in vivo vascular analysis we used AngioTool64 version 0.6a<br>- For IVIS analysis Living Image 4.5 (Perkin Elmer).

For manuscripts utilizing custom algorithms or software that are central to the research but not yet described in published literature, software must be made available to editors and reviewers. We strongly encourage code deposition in a community repository (e.g. GitHub). See the Nature Portfolio guidelines for submitting code & software for further information.

Data

Policy information about availability of data

All manuscripts must include a data availability statement. This statement should provide the following information, where applicable:

- Accession codes, unique identifiers, or web links for publicly available datasets
- A description of any restrictions on data availability
- For clinical datasets or third party data, please ensure that the statement adheres to our policy

Source data are provided with this paper.

Human research participants

Policy information about studies involving human research participants and Sex and Gender in Research.

Reporting on sex and gender	N/A
Population characteristics	N/A
Recruitment	N/A
Ethics oversight	N/A

Note that full information on the approval of the study protocol must also be provided in the manuscript.

Field-specific reporting

Please select the one below that is the best fit for your research. If you are not sure, read the appropriate sections before making your selection.

☒ Life sciences ☐ Behavioural & social sciences ☐ Ecological, evolutionary & environmental sciences

For a reference copy of the document with all sections, see nature.com/documents/nr-reporting-summary-flat.pdf

Life sciences study design

All studies must disclose on these points even when the disclosure is negative.

Sample size	All sample size are indicated in the figure legends, an the sample size were sufficient to conduct reasonable statistical analyses where applicable. For in vivo experiments to establish a reasonable minimum sample size we used G-power v3.1.9.7 (Heinrich-Heine-Universität Düsseldorf) with One way-Anova analysis between groups, with 80% of power and 5% of alpha error. From the analysis we obtained n=8 mice as minimum sample size for VEP,KC and DL groups. Liver, as pilot group, was not included in the original sample size evaluation. This is due to the different number of NPIs implanted that modify a priori the level of comparison.
Data exclusions	Based on our pubblications (PMID: 23751893; PMID: 17264813; PMID: 30735895) and other in Nature Publishing group in beta cell replacement (PMID: 25893782; PMID: 31582751) we set up a stringent diabetes induction glycemia level to avoid the peristency of a residual beta cell mass in recipient mice. In induced diabetic mouse models, only mice with blood glucose level > 450 mg/dl for two consecutive measurements were used.
Replication	All detail on biological and technical replicates are provided in the text and/or figure legends . Results were consistent across independent experiments
Randomization	For ex vivo experiment experimental group, cells were randomly distributed from each batch within experimental groups. For all animal experiments, mice were randomly allocated into each experimental group
Blinding	Investigator performing tissue collections and analysis were blind to group allocation.

Reporting for specific materials, systems and methods

We require information from authors about some types of materials, experimental systems and methods used in many studies. Here, indicate whether each material, system or method listed is relevant to your study. If you are not sure if a list item applies to your research, read the appropriate section before selecting a response.

Materials & experimental systems

n/a	Involved in the study	
☐	☒	Antibodies
☒	☐	Eukaryotic cell lines
☒	☐	Palaeontology and archaeology
☐	☒	Animals and other organisms
☒	☐	Clinical data
☒	☐	Dual use research of concern

Methods

n/a	Involved in the study	
☒	☐	ChIP-seq
☐	☒	Flow cytometry
☒	☐	MRI-based neuroimaging

Antibodies

Antibodies used	For flow citometry: anti-CD31-APC (1:100, Immunotools, 21270316), anti-CD34-PE (1:100, Immunotools, 21270344), anti-KDR-PE (VEGFR-2) (1:100, Miltenyi Biotech, 130-098-905), anti-TIE-2-APC (1:100, Miltenyi Biotech, 130-101-606), and anti-VE-Chaderin-PE (1:100, Miltenyi Biotech, 130-100-716)<br>For immunofluorescence: human CD31 (1:40, DAKO, M0823, clone: JC70A), human vWF (1:200, DAKO, A0082, polyclonal), human VE-Cadherin (1:40, R&D system,AF938, polyclonal), human VEGF-R2 (1:200, Cell Signaling, 2479S, polyclonal), pig Insulin (1:200; DAKO, A0564, polyclonal), pig Chromogranin A (1:200, Abcam, AB15160, polyclonal), mouse CD31(1:300, Biolegend, 102501, clone: MEC13.3), mouse CD11b-PE (1:100, Biolegend, 101208, clone: M1/70), mouse LY6G-AF647(1:100, Biolegend, 127610, clone: 1A8), mouse anti-tubulin β3-AF647 (1:100 Biolegend, 801210, clone: TUJ1), anti-pig somatostatin (H-11) (1:100 Santa Cruz Biotechnology, Sc-74556, clone H-11), anti-pig glucagon (1:200 Invitrogen, PA5-83353, polyclonal).
Validation	All antibodies used in our study are commercially available and validated by manufacturer.<br>http://www.immunotools.de/html/datas-apc/21270316.pdf; http://www.immunotools.de/html/datas-pe/21270344.pdf; https://www.miltenyibiotec.com/IT-en/products/macs-antibodies/antibody-validation.html#gref; https://www.agilent.com/en/product/dako-omnis-solution-for-ihc-ish/primary-antibodies-for-dako-omnis; https://www.rndsystems.com/products/antibodies; https://www.cellsignal.com/about-us/cst-antibody-validation-principles; https:// https://www.abcam.com/primary-antibodies/how-we-validate-our-antibodies; https://www.biolegend.com/en-us/bio-bits/highly-specific-validated-antibodies; https://www.scbt.com/p/somatostatin-antibody-h-11?requestFrom=search; https://www.thermofisher.com/it/en/home/life-science/antibodies/invitrogen-antibody-validation.html

Animals and other research organisms

Policy information about studies involving animals; ARRIVE guidelines recommended for reporting animal research, and Sex and Gender in Research

Laboratory animals	6-8 weeks female NSG mice (22-25gr) purchased from Charles River laboratory. The mice were kept in 12hour/12hour light/dark cycles, at 21.5°C +/- 1.5°C temperature and 55% +/- 15 humidity. 6-8 weeks old male Lewis rats (175-200gr) were purchased from Charles River laboratory. The rats were kept in 12hour/12hour light/dark cycles, at 21.5°C +/- 1.5°C temperature and 55% +/- 15 humidity. 1-7 days German Landrace for WT or CAG-iRFP piglets were raised at the Center for Innovative Medical Models (CiMM), LMU Munich. Piglets were housed in a farrowing pen together with the mother, and a heated nest were offered to the piglets. Housing and pig breeding and handling procedures were approved by district governments of Upper Bavaria, Germany and were conducted in accordance with the German Animal Welfare Acts
Wild animals	No wild animals were used in the study.
Reporting on sex	Female NSG, male Lewis rat. Sex distribution of pancreas donor piglets were 66% males and 34% females for WT piglets and 44% males and 56% females for CAG-iRFP transgenic piglets. To our best knowledge, gender of pancreas donor piglets do not have any impact on NPI isolation and graft function
Field-collected samples	The study didn't involve animals collected from the field.
Ethics oversight	All animal procedures were performed according to protocols approved by the Animal Care and Use Committee (IACUC) of San Raffaele Scientific Institute and district governments of Upper Bavaria, Germany and were conducted in accordance with the German Animal Welfare Act

Note that full information on the approval of the study protocol must also be provided in the manuscript.

Flow Cytometry

Plots

Confirm that:

☒ The axis labels state the marker and fluorochrome used (e.g. CD4-FITC).

☒ The axis scales are clearly visible. Include numbers along axes only for bottom left plot of group (a 'group' is an analysis of identical markers).

☒ All plots are contour plots with outliers or pseudocolor plots.

☒ A numerical value for number of cells or percentage (with statistics) is provided.

Methodology

Sample preparation	BOEC cells from healthy donor subject were used for flow citometry analysis. As reported in Methods sections BOECs, once obtained as single cell suspension, were stained for 30 min at 4°C in a dark place with Live Dead Pacific Blue dye (Life technologies) using 1 µL of dye every 1x106 cells resuspended in 1 mL of PBS. Cells are stained for 20 minutes with primary antibodies, listed in the previous sections, in dark at 4°C. Samples are fixed with 200 µL cytofix/cytoperm buffer (ThermoFisher) for 20 minutes. Before every step, sample were washed with FACS Buffer and centrifugated at 1200 rpm for 8 minutes.
Instrument	FACSCantoII instrument (BD Biosciences)
Software	FlowJo 10.6.2
Cell population abundance	Cell sorting was not performed
Gating strategy	Samples were firstly gated to eliminate debris (by FSC Vs SSC), then gated on single cells (FSC-W Vs FSC-H) following dead cell exclusion (as described in the method section) and analysis for the expression of specific markers. Gating strategy is shown in Supplementary Fig.1.

☒ Tick this box to confirm that a figure exemplifying the gating strategy is provided in the Supplementary Information.
